# Supplementary material for: Substrate preferences, phylogenetic and biochemical properties of proteolytic bacteria present in the digestive tract of Nile tilapia (Oreochromis niloticus)
Source: AIMS Microbiol. 2021 Dec 23;7(4):528–45. doi: 10.3934/microbiol.2021032 (PMC8712536; doi:10.3934/microbiol.2021032)
Supplement: Supplementary file 1 [file microbiol-07-04-032-s001.pdf]

AIMS Microbiology

AIMS Microbiology, 7(4): 528–545.
DOI: 10.3934/microbiol.2021032
Received: 22 October 2021
Accepted: 20 December 2021
Published: 23 December 2021

http://www.aimspress.com/journal/microbiology

Research article

Substrate preferences, phylogenetic and biochemical properties of proteolytic bacteria present in the digestive tract of Nile tilapia (*Oreochromis niloticus*)

Tanim Jabid Hossain[1,2,*], Mukta Das[1,2], Ferdausi Ali[3], Sumaiya Islam Chowdhury[1,2] and Subrina Akter Zidny[1,2]

[1] Department of Biochemistry and Molecular Biology, University of Chittagong, Chattogram 4331, Bangladesh
[2] Biochemistry and Pathogenesis of Microbes Research Group, Chattogram 4331, Bangladesh
[3] Department of Microbiology, University of Chittagong, Chattogram 4331, Bangladesh

* **Correspondence:** Email: tanim.bmb@gmail.com.

Supplementary

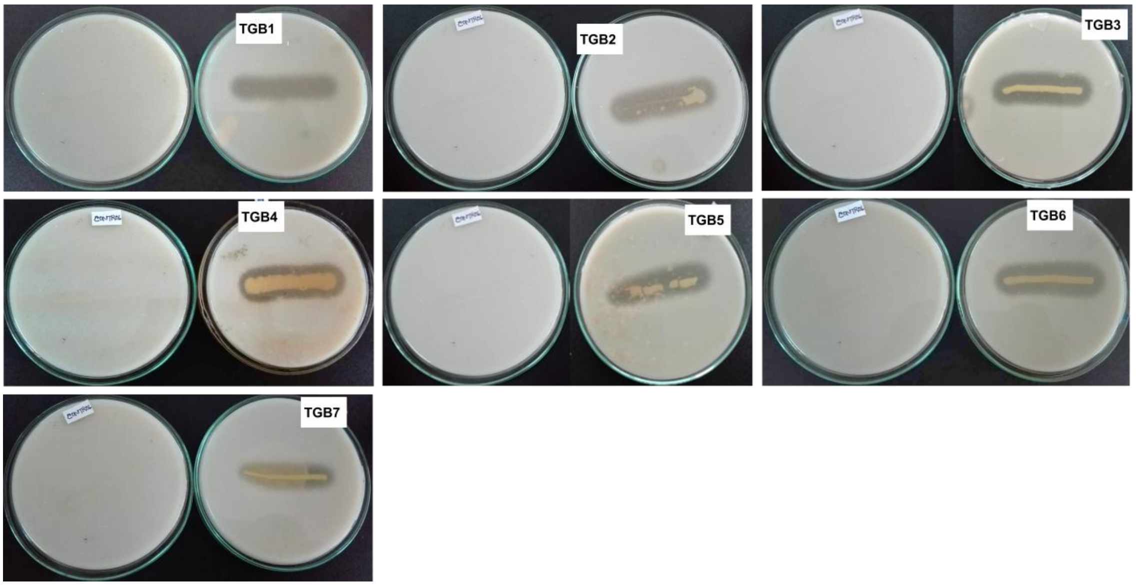

Figure S1. Screening for proteolytic activity of the isolates, indicated by clear zones around the colonies grown on media containing casein. For each isolate, the plate at the left shows negative control in which bacteria without proteolytic activity was streaked on the media.

AIMS Press

© 2021 the Author(s), licensee AIMS Press. This is an open access article distributed under the terms of the Creative Commons Attribution License (http://creativecommons.org/licenses/by/4.0)
